# Prediction of conformational changes by single mutation in the hepatitis B virus surface antigen (HBsAg) identified in HBsAg-negative blood donors

**DOI:** 10.1186/1743-422X-7-326

**Published:** 2010-11-18

**Authors:** Susan I Ie, Meta D Thedja, Martono Roni, David H Muljono

**Affiliations:** 1 Eijkman Institute for Molecular Biology, Jl. Diponegoro 69, Jakarta, Indonesia

## Abstract

**Background:**

Selection of hepatitis B virus (HBV) by host immunity has been suggested to give rise to variants with amino acid substitutions at or around the *'a' *determinant of the surface antigen (HBsAg), the main target of antibody neutralization and diagnostic assays. However, there have never been successful attempts to provide evidence for this hypothesis, partly because the 3 D structure of HBsAg molecules has not been determined. Tertiary structure prediction of HBsAg solely from its primary amino acid sequence may reveal the molecular energetic of the mutated proteins. We carried out this preliminary study to analyze the predicted HBsAg conformation changes of HBV variants isolated from Indonesian blood donors undetectable by HBsAg assays and its significance, compared to other previously-reported variants that were associated with diagnostic failure.

**Results:**

Three HBV variants (T123A, M133L and T143M) and a wild type sequence were analyzed together with frequently emerged variants T123N, M133I, M133T, M133V, and T143L. Based on the Jameson-Wolf algorithm for calculating antigenic index, the first two amino acid substitutions resulted in slight changes in the antigenicity of the *'a' *determinant, while all four of the comparative variants showed relatively more significant changes. In the pattern T143M, changes in antigenic index were more significant, both in its coverage and magnitude, even when compared to variant T143L. These data were also partially supported by the tertiary structure prediction, in which the pattern T143M  showed larger shift in the HBsAg second loop structure compared to the others.

**Conclusions:**

Single amino acid substitutions within or near the *'a' *determinant of HBsAg may alter antigenicity properties of variant HBsAg, which can be shown by both its antigenic index and predicted 3 D conformation. Findings in this study emphasize the significance of variant T143M, the prevalent isolate with highest degree of antigenicity changes found in Indonesian blood donors. This highlights the importance of evaluating the effects of protein structure alterations on the sensitivity of screening methods being used in detection of ongoing HBV infection, as well as the use of vaccines and immunoglobulin therapy in contributing to the selection of HBV variants.

## Background

Hepatitis B Virus (HBV), the etiology of hepatitis B, is a DNA virus that replicates via an RNA intermediate [1]. It has a small partially double-stranded DNA genome of approximately 3.2 kilobases that contains four overlapping open reading frames, including one that encodes for the hepatitis B surface antigen (HBsAg) protein [1]. Diagnosis and screening of HBV infection is most commonly done by detection of the HBsAg by means of antibody-based assays [2]. These assays target the *'a' *determinant, the highly homologous region within HBsAg, which is also used as the main target of antibody generated by hepatitis B vaccines [2]. However, there have been reports on the failure of these assays in detecting HBsAg in infected individuals, which include inactive HBV carriers, vaccinated children born to mothers with HBV infection, and liver transplant recipients treated with hepatitis B immunoglobulin (HBIg) therapy [3-5].

Recognition of the *'a' *determinant by antibody against HBsAg (anti-HBs) depends on its 3 D conformation, which also relies on the amino acid sequence of the regions flanking the *'a' *determinant [6,7]. To date, there have never been successful attempts on crystallizing native HBsAg molecules for structure determination purposes. Tertiary structures of HBsAg have not been fully determined, aside from its nature as a membrane spanning protein with four trans-membrane helices and a major hydrophilic region that is exposed on the surface of the virus [7,8]. It is of interest to be able to predict the tertiary structure of HBsAg solely from its primary amino acid sequence, because pathogen recognition by the host immune system is mainly based on protein-protein interaction, which depends on the conformation of the interacting proteins. We carried out this preliminary study to analyze the prediction of HBsAg conformation changes as caused by variations in the S gene of HBV isolated from Indonesian HBsAg-negative blood donors in comparison with variants frequently reported from various regions of the world. The results of this study may contribute in better understanding the host-pathogen interaction as well as paving the way to develop better techniques in designing diagnostic tools and vaccine candidates for hepatitis B.

## Materials and methods

### Sample selection and preparation

This study is part of a larger project investigating the main transfusion-transmitted infections including hepatitis B in regular blood donors by the Indonesian Red Cross in two cities of Indonesia, Medan of Sumatra and Solo of Java islands. Previous study by Thedja *et al*., 2010 showed that HBV DNA was detected in 25 (8.1%) of 309 HBsAg-negative blood donors [9]. HBV DNA in the blood donors' samples was undetectable by quantitative PCR and detectable only in the second-round of nested PCR, which was capable of detecting HBV DNA at titres lower than the detection limit of the Cobas-Taqman 48 Real-Time PCR (Roche Molecular System, Branchburg, NJ, USA), 6 IU/mL [9,10]. The sequences of HBV DNA isolated in the study had been deposited in GenBank under Accession Nos. EF507434-EF507475 and HM116516-HM116533. To analyze the HBsAg conformation changes resulted from variations in the S gene, we first aligned the translated nucleotide sequences of HBV isolated from the Indonesian HBsAg-negative blood donors with a wild type reference (M54923; genotype B/*adw*) retrieved from GenBank [11], using BioEdit Sequence Alignment Editor Ver. 7.0.5.2 software [12]. Next, we searched for more HBV variants reported in association with medical and public health issues (problems in diagnostic assays and/or escape to vaccine/HBIg therapy) from published articles and GenBank database, focusing on variants with substitutions at the corresponding amino acid positions. Totally, an additional 5 sequences were retrieved and analyzed for their antigenic index calculation.

### Prediction of antigenicity

Translated HBsAg sequences that contain mutations were analyzed with Jameson-Wolf algorithm in the Lasergene Protean v8.1 program (DNASTAR Inc., Madison, WI) to predict the antigenic index of each consensus sequence. This algorithm integrates several parameters to calculate the antigenicity of the sequence based on the characteristics of its primary amino acid chain: hydrophilicity (Hopp-Woods), surface probability (Emini), flexibility of the protein backbone (Karplus-Schulz), and secondary structure prediction (Chou-Fasman and Garnier) using the following equation [13]:

$${\text{A}}_{i}\;=\;\sum_{i=1}^{N}0.\text{3}\left({\text{H}}_{i}\right)+0.\text{15}\left({\text{S}}_{i}\right)+0.\text{15}\left({\text{F}}_{i}\right)+0.\text{2}\left({\text{CF}}_{i}\right)+0.\text{2}\left({\text{RG}}_{i}\right)$$

with regions of positive A*_i _*value clusters indicate possible antigenic determinants.

### Tertiary structure prediction

Based on structural alignment using Template Identification tool from Swiss-Model by InterPro Scan, BLASTP 2.2.9, PSI-BLAST, and HHSEARCH v. 1.5.01 software [14-17], no template structure was found in ExPDB template library for the 226-amino-acid-long HBsAg [18]. Therefore, tertiary structures of the HBsAg variants found in Indonesian blood donor samples were predicted using free modelling, or often termed as '*ab initio*' or '*de novo*' modelling [19]. In this study, we used I-TASSER method, a protein structure modelling approach based on an algorithm consists of consecutive steps of threading, fragment assembly, and iteration to obtain structure with the lowest energy as described previously [20-22]. All structure predictions of wild type reference sequence and the variants were predicted separately using individual I-TASSER queries, and visualized using DeepView/Swiss-PdbViewer [23].

## Results

### Characterization of HBV mutants

**Figure 1:**
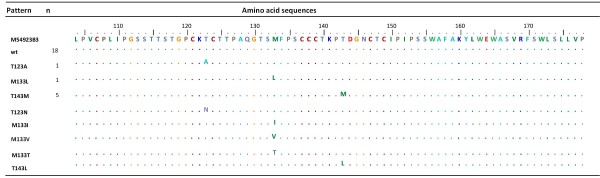
**Alignment of amino acid sequences of HBV isolates in Indonesian blood donors with frequently-reported variants associated with failure of diagnostic assays**. Three amino acid substitutions were identified in 7 HBV isolates in blood donors: Pattern 1, T123A, in one isolate; Pattern 2, M133L, in one isolate; Pattern 3, T143M, in five isolates. HBV DNA isolated from the remaining 18 samples showed wild type (wt) sequences with no amino acid substitution. Consensus of each of the three single mutation patterns and wt were aligned with five known variants frequently associated with problems in diagnostic assays and/or escape to vaccine/HBIg therapy: T123N, M133I, M133T, M133V, and T143L, together with M54923 sequence (genotype B/*adw*) retrieved from GenBank as a reference.

Sequencing of partial HBV surface gene of the clones derived from 25 HBV DNA positive samples [9] showed nucleotide substitutions in 7 samples: A521G in one sample, A551T and A562G in one sample, and C582T in five samples. Of the four nucleotide substitutions, three single mutation patterns (T123A, M133L and T143M) of HBV surface protein were observed, while A562G was found to be a silent mutation. These mutation positions corresponded with those of five isolates known to be associated with problem in diagnostic assays and/or escape to vaccine/HBIg therapy: T123N, M133I, M133T, M133V, and T143L [5,24-29] (Figure 1). The remaining 18 (72%) samples did not show any nucleotide substitutions [9].

### Prediction of antigenicity

Prediction of antigenic index of mutant sequences notably revealed altered antigenicity at and around the sites of amino acid substitutions compared to the wild type sequence (Table 1). In T123A substitution, several amino acids were affected by this single substitution. Antigenic index values of four amino acids at the region around amino acid position 123 was altered between -0.4 to +0.2 in magnitude. In contrast, only a small antigenicity change was detected (from -0.2 to -0.05) at the single amino acid site of M133L substitution. Most significant changes were observed in the T143M substitution. In this last pattern, antigenic index of the residues at position 143 and up to 5 amino acids both upstream and downstream of this site were observed to be altered between -1.07 to +0.62 in magnitude. These antigenic index changes were grouped into collectively negative alterations - i.e. more hydrophobic characteristics - upstream of the Met at 143, and relatively positive or more hydrophilic downstream. In comparison, T123N and M133I/V/T missed in diagnostic assays presented more altered antigenic index profiles, while T143L showed similar if not lesser degree of changes (Table 1).

**Table 1:** The Jameson-Wolf antigenicity index prediction of HBsAg within amino acid 118 - 160

Position	M54923		Pattern T123A*		Pattern T123N**		Pattern M133L*		Pattern M133I**		Pattern M133T**		Pattern M133V**		Pattern T143M*		Pattern T143L**	
	
	Residue	Antigenic Index	Residue	Antigenic Index	Residue	Antigenic Index	Residue	Antigenic Index	Residue	Antigenic Index	Residue	Antigenic Index	Residue	Antigenic Index	Residue	Antigenic Index	Residue	Antigenic Index
110	Ile	-0.2	Ile	-0.2	Ile	-0.2	Ile	-0.2	Ile	-0.2	Ile	-0.2	Ile	-0.2	Ile	-0.2	Ile	-0.2

111	Pro	-0.05	Pro	-0.05	Pro	-0.05	Pro	-0.05	Pro	-0.05	Pro	-0.05	Pro	-0.05	Pro	-0.05	Pro	-0.05

112	Gly	0.35	Gly	0.35	Gly	0.35	Gly	0.35	Gly	0.35	Gly	0.35	Gly	0.35	Gly	0.35	Gly	0.35

113	Ser	0.8	Ser	0.8	Ser	0.8	Ser	0.8	Ser	0.8	Ser	0.8	Ser	0.8	Ser	0.8	Ser	0.8

114	Ser	1	Ser	1	Ser	1	Ser	1	Ser	1	Ser	1	Ser	1	Ser	1	Ser	1

115	Thr	0.4	Thr	0.4	Thr	0.4	Thr	0.4	Thr	0.4	Thr	0.4	Thr	0.4	Thr	0.4	Thr	0.4

116	Thr	1.05	Thr	1.05	Thr	***0.8***	Thr	1.05	Thr	1.05	Thr	1.05	Thr	1.05	Thr	1.05	Thr	1.05

117	Ser	1.3	Ser	1.3	Ser	***1.05***	Ser	1.3	Ser	1.3	Ser	1.3	Ser	1.3	Ser	1.3	Ser	1.3

118	Thr	1.2	Thr	1.2	Thr	***0.95***	Thr	1.2	Thr	1.2	Thr	1.2	Thr	1.2	Thr	1.2	Thr	1.2

119	Gly	2.05	Gly	2.05	Gly	***1.6***	Gly	2.05	Gly	2.05	Gly	2.05	Gly	2.05	Gly	2.05	Gly	2.05

120	Pro	2.5	Pro	2.5	Pro	***2.05***	Pro	2.5	Pro	2.5	Pro	2.5	Pro	2.5	Pro	2.5	Pro	2.5

121	Cys	2.25	Cys	2.25	Cys	***2.5***	Cys	2.25	Cys	2.25	Cys	2.25	Cys	2.25	Cys	2.25	Cys	2.25

122	Lys	2	Lys	***1.6***	Lys	***2.25***	Lys	2	Lys	2	Lys	2	Lys	2	Lys	2	Lys	2

**123**	Thr	0.35	**Ala**	***0.4***	**Asn**	***2***	Thr	0.35	Thr	0.35	Thr	0.35	Thr	0.35	Thr	0.35	Thr	0.35

124	Cys	0.25	Cys	***0.15***	Cys	***1.5***	Cys	0.25	Cys	0.25	Cys	0.25	Cys	0.25	Cys	0.25	Cys	0.25

125	Thr	0.45	Thr	***0.65***	Thr	***0.9***	Thr	0.45	Thr	0.45	Thr	0.45	Thr	0.45	Thr	0.45	Thr	0.45

126	Thr	0.25	Thr	0.25	Thr	0.25	Thr	0.25	Thr	0.25	Thr	0.25	Thr	0.25	Thr	0.25	Thr	0.25

127	Pro	0.4	Pro	0.4	Pro	0.4	Pro	0.4	Pro	0.4	Pro	0.4	Pro	0.4	Pro	0.4	Pro	0.4

128	Ala	0.8	Ala	0.8	Ala	0.8	Ala	0.8	Ala	0.8	Ala	0.8	Ala	0.8	Ala	0.8	Ala	0.8

129	Gln	0.8	Gln	0.8	Gln	***0.4***	Gln	0.8	Gln	0.8	Gln	0.8	Gln	0.8	Gln	0.8	Gln	0.8

130	Gly	0.65	Gly	0.65	Gly	0.65	Gly	0.65	Gly	***0.25***	Gly	***0.8***	Gly	***0.25***	Gly	0.65	Gly	0.65

131	Thr	0.35	Thr	0.35	Thr	0.35	Thr	0.35	Thr	***-0.05***	Thr	0.35	Thr	***-0.05***	Thr	0.35	Thr	0.35

132	Ser	0.35	Ser	0.35	Ser	0.35	Ser	0.35	Ser	***-0.05***	Ser	***0.65***	Ser	***-0.05***	Ser	0.35	Ser	0.35

**133**	Met	-0.2	Met	-0.2	Met	-0.2	**Leu**	***-0.05***	**Ile**	***-0.6***	**Thr**	***0.8***	**Val**	***-0.45***	Met	-0.2	Met	-0.2

134	Phe	-0.2	Phe	-0.2	Phe	-0.2	Phe	-0.2	Phe	-0.2	Phe	***-0.05***	Phe	-0.2	Phe	-0.2	Phe	-0.2

135	Pro	0.2	Pro	0.2	Pro	0.2	Pro	0.2	Pro	0.2	Pro	***0.35***	Pro	0.2	Pro	0.2	Pro	0.2

136	Ser	0.2	Ser	0.2	Ser	0.2	Ser	0.2	Ser	0.2	Ser	0.2	Ser	0.2	Ser	0.2	Ser	0.2

137	Cys	0.2	Cys	0.2	Cys	0.2	Cys	0.2	Cys	0.2	Cys	0.2	Cys	0.2	Cys	0.2	Cys	0.2

138	Cys	0.64	Cys	0.64	Cys	0.64	Cys	0.64	Cys	0.64	Cys	0.64	Cys	0.64	Cys	***0.3***	Cys	***0.3***

139	Cys	1.18	Cys	1.18	Cys	1.18	Cys	1.18	Cys	1.18	Cys	1.18	Cys	1.18	Cys	***0.5***	Cys	***0.8***

140	Thr	1.27	Thr	1.27	Thr	1.27	Thr	1.27	Thr	***1.67***	Thr	1.27	Thr	1.27	Thr	***0.41***	Thr	***0.7***

141	Lys	2.36	Lys	2.36	Lys	2.36	Lys	2.36	Lys	2.36	Lys	2.36	Lys	2.36	Lys	***1.47***	Lys	***1.75***

142	Pro	3.4	Pro	3.4	Pro	3.4	Pro	3.4	Pro	3.4	Pro	3.4	Pro	3.4	Pro	***2.33***	Pro	***2.6***

**143**	Thr	2.86	Thr	2.86	Thr	2.86	Thr	2.86	Thr	2.86	Thr	2.86	Thr	2.86	**Met**	***2.74***	**Leu**	***3***

144	Asp	2.57	Asp	2.57	Asp	2.57	Asp	2.57	Asp	2.57	Asp	2.57	Asp	2.57	Asp	***3.1***	Asp	***2.45***

145	Gly	1.93	Gly	1.93	Gly	1.93	Gly	1.93	Gly	1.93	Gly	1.93	Gly	1.93	Gly	***2.49***	Gly	***2.15***

146	Asn	1.59	Asn	1.59	Asn	1.59	Asn	1.59	Asn	1.59	Asn	1.59	Asn	1.59	Asn	***1.58***	Asn	***1.25***

147	Cys	0.1	Cys	0.1	Cys	0.1	Cys	0.1	Cys	0.1	Cys	0.1	Cys	0.1	Cys	***0.72***	Cys	***0.4***

148	Thr	-0.6	Thr	-0.6	Thr	-0.6	Thr	-0.6	Thr	-0.6	Thr	-0.6	Thr	-0.6	Thr	***-0.29***	Thr	-0.6

149	Cys	-0.6	Cys	-0.6	Cys	-0.6	Cys	-0.6	Cys	-0.6	Cys	-0.6	Cys	-0.6	Cys	-0.6	Cys	-0.6

150	Ile	-0.6	Ile	-0.6	Ile	-0.6	Ile	-0.6	Ile	-0.6	Ile	-0.6	Ile	-0.6	Ile	-0.6	Ile	-0.6

151	Pro	-0.6	Pro	-0.6	Pro	-0.6	Pro	-0.6	Pro	-0.6	Pro	-0.6	Pro	-0.6	Pro	-0.6	Pro	-0.6

152	Ile	-0.45	Ile	-0.45	Ile	-0.45	Ile	-0.45	Ile	-0.45	Ile	-0.45	Ile	-0.45	Ile	-0.45	Ile	-0.45

153	Pro	-0.05	Pro	-0.05	Pro	-0.05	Pro	-0.05	Pro	-0.05	Pro	-0.05	Pro	-0.05	Pro	-0.05	Pro	-0.05

154	Ser	0.35	Ser	0.35	Ser	0.35	Ser	0.35	Ser	0.35	Ser	0.35	Ser	0.35	Ser	0.35	Ser	0.35

155	Ser	0.35	Ser	0.35	Ser	0.35	Ser	0.35	Ser	0.35	Ser	0.35	Ser	0.35	Ser	0.35	Ser	0.35

156	Trp	-0.2	Trp	-0.2	Trp	-0.2	Trp	-0.2	Trp	-0.2	Trp	-0.2	Trp	-0.2	Trp	-0.2	Trp	-0.2

157	Ala	-0.6	Ala	-0.6	Ala	-0.6	Ala	-0.6	Ala	-0.6	Ala	-0.6	Ala	-0.6	Ala	-0.6	Ala	-0.6

158	Phe	-0.6	Phe	-0.6	Phe	-0.6	Phe	-0.6	Phe	-0.6	Phe	-0.6	Phe	-0.6	Phe	-0.6	Phe	-0.6

159	Ala	-0.6	Ala	-0.6	Ala	-0.6	Ala	-0.6	Ala	-0.6	Ala	-0.6	Ala	-0.6	Ala	-0.6	Ala	-0.6

160	Lys	-0.6	Lys	-0.6	Lys	-0.6	Lys	-0.6	Lys	-0.6	Lys	-0.6	Lys	-0.6	Lys	-0.6	Lys	-0.6

*Variants found in Indonesian blood donor; **Variants frequently associated with problems in diagnostic assays and/or escape to vaccine/HBIg therapy. Residues with substitutions and their positions are shown in **bold**. Altered antigenicity index of affected residues in each substitution pattern are shown in **bold **and *italics*: T123A alters four consecutive residues (aa 122-125); M133L alters the antigenic index of position 133 only; T123N, M133I/T/V, and T143L cause relatively extensive antigenic index changes in 11, 5, 5, 4, and 10 residues, respectively; T143 M shows the most significant changes in the antigenic profile of HBsAg between residues 138 to 148.

### Tertiary structure prediction

**Figure 2:**
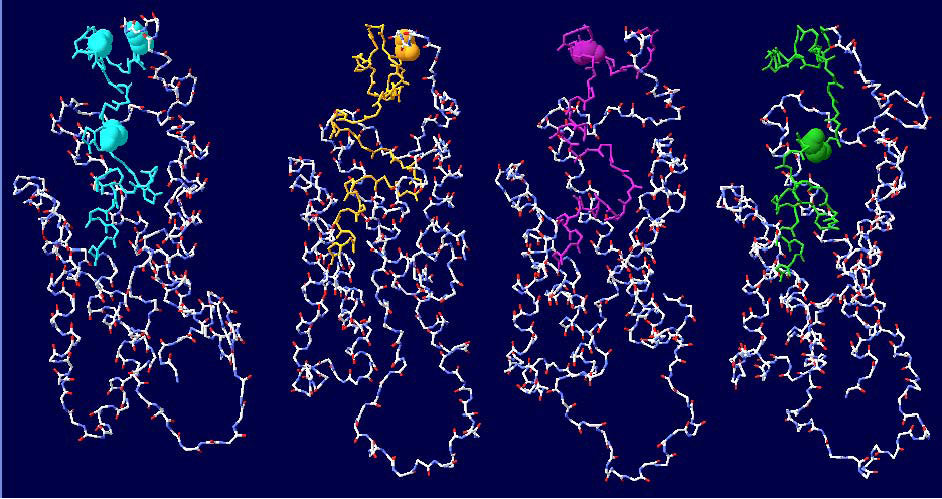
**Comparison of tertiary structure prediction**. Tertiary structure prediction of M54923 (reference sequence), T123A, M133L, and T143M mutants. The *'a' *determinant is shown in blue, yellow, magenta, and green, respectively, while residues of importance are labelled with the side chains shown.

The tertiary structure prediction of each variant isolated from Indonesian blood donors differed slightly from the wild type reference sequence, particularly in the *'a' *determinant region (Figure 2). The structure of the mainframe, which consisted mainly of helical structures, tended to be retained in all sequences, while the loop structures, including the *'a' *determinant, tended to differ slightly between these sequences. In pattern T123A, the loop containing the *'a' *determinant seemed to shift slightly compared to the reference wild-type. Although the side chain of Ala did not differ much in its orientation and position, the remainder of the loop shifted noticeably, as could be seen in the difference of the coiling and bends of the loop that made the contour of the *'a' *determinant against the cavity in the mainframe helices. Similar shift in loop structure was observed in pattern M133L, as could be shown in the different orientation of Leu side chain in position 133 compared to Met side chain in the wild-type. The pattern T143M, on the other hand, besides showing differentially-oriented side chain of Met, also showed significant changes in larger part of the loop. Larger region of the loop N-terminally of position 143 seemed to uncoil, while the loop positioned C-terminally of residue 143 bent closer toward the mainframe cavity compared to the reference structure.

## Discussion

HBV mechanism of replication includes an RNA intermediate that is reverse-transcribed into DNA by error-prone RNA polymerase [30]. This process results in a high mutation rate of approximately 1.4-3.2 × 10^-5 ^substitutions/site/year for the whole genome and even higher for the surface gene [30,31]. This allows the virus to evolve within a chronically infected individual to form a naturally occurring quasi-species pool of HBV variants [5,29]. In regions with high HBV endemicity, the relatively high rate of viral transmission might provide more opportunities for super-infection and multiple infections to occur, which would result in increased number of variants circulating within individuals as well as in the population [2,32]. The composition of variants in the viral population is maintained by its environment. Variants better suited to the host environment would prevail and dominate the population [33]. In such cases, environmental changes induced by either natural immune response, vaccine-induced or therapeutic immunoglobulin (HBIg), or even anti-viral therapy may select for variants that can evade these protective measures, particularly those exhibiting mutation-induced conformational changes at the antigenic *'a' *determinant of its surface antigen [2,3,5]. Selection of variants is usually indicated by certain serological markers, such as isolated anti-HBc, co-occurrence of both HBsAg and anti-HBs, and inconsistent HBsAg assay results [34]. The presence of these variants poses potential threat to the success of vaccination and supply of safe blood products due to the possible evasion from vaccine-generated antibody and poor detection by the available diagnostic assays [6].

Numerous studies have shown that three dimensional conformations of proteins contribute toward their biological functions as well as their interactions with other molecules [35,36]. Substitutions of key amino acid residues may affect the stability and structure of a protein, altering its properties and interactions with other particles. Protein modelling of HBsAg variants might give insight into the structural basis of HBV variation at the molecular level, and how it affects the HBsAg recognition by its specific antibody.

Substitutions of Thr 123, Met 133 and Thr 143 into other amino acid residues as found in this study had been described in relation to failure of HBIg therapy and problems in detection assays [5,24-29,37,38]. The outcome of these substitutions is related to the site of mutation and the property of the respective amino acid, which is also observed in the mutants found in this study. Thr123, although located upstream of the *'a' *determinant, is in close proximity to the Cys 124 residue responsible for maintaining the integrity of HBsAg antigenic loop. There had been reports of insertions between Cys residues 121 and 124 that reduced or abolished bindings by monoclonal antibodies [39,40]. Furthermore, in a study by Chen *et al*., the preservation of Thr at residue 123 seemed to be an important factor in the recognition of one of the *'a' *determinant epitopes by monoclonal anti-HBs [7]. Hence, the substitution site is important because it may disturb the disulphide bonds, leading to the alteration of loop conformation and decrease or loss of neutralizing antibody binding.

The other two mutation sites, Met 133 and Thr 143, are located within the first (aa 124-137) and second (aa 139-147) antigenic loops of the *'a' *determinant, respectively [7,8,41]. Ample reports on substitutions within these two regions had been published [5,24,26,27,37,40-43], as the *'a' *determinant is known as the main antibody recognition site of HBsAg. Mutations at these regions would predictably affect the loop conformation and causes problems of escape mutants and diagnostic failure.

As of the property of each amino acid, protein is a macromolecule made of monomeric amino acids. Each amino acid has distinct properties attributable to its side-chain, and the structure of a protein is dependent on the composition of its amino acids [44]. Therefore, differences in amino acid properties might contribute to the changes in the structure of the *'a' *determinant loop. Methionine, Alanine, Leucine, Isoleucine, and Valine are amino acids with non-polar, aliphatic side chains, while Threonine and Aspargine have a polar although uncharged side chain (-CH(CH_3_)-OH and -CH_2_-CO-NH_2 _groups). Within the non-polar, aliphatic amino acids themselves, there are differences in the length and bulkiness of the side chain; alanine has a methyl group (-CH_3_), valine with iso-propyl group (-CH(CH_3_)-CH_3_), leucine with iso-butyl group (-CH_2_-CH(CH_3_)-CH_3_), isoleucine with 2°-butyl group (-CH(CH_3_)- CH_2_-CH_3_) and methionine with a methyl-ethyl-sulphide group (-CH_2_-CH_2_-S-CH_3_). These slight differences in the amino acid properties may affect the tertiary structure of the protein, as different polarity determines the hydrophobicity of the residue, while differences in length and bulkiness of the side chain may influence the steric hindrance between neighbouring residues [44].

The degree of changes in antigenicity profile was highest in pattern T143M , followed closely by T123N and T143L, then lesser changes in M133I/T/V as well as T123A and M133L. M133L mutant showed the least significant changes, probably because it is located in less-antigenic first loop [41], and also because both Met and Leu are non-polar residues with similar bulkiness of their side-chains. T123A mutant, on the other hand, involved changes from a polar Thr into a non-polar and slightly smaller Ala. Although it may affect the conformation by means of influencing the disulphide bond, the effect would be minimized because of the nature and size of Ala. The trend in M133I/T/V can also be correlated with the differential amino acid properties, with similar changes between M133I and M133V that involve similarly-sized non polar Met, Ile, and Val; and slightly more significant antigenic alteration in M133T, in which there is a change from Met to polar Thr. Marked changes were also observed in T123N and T143L substitutions, which might be caused by both the shift from slightly small, polar Thr into either larger, more polar Asp or bulkier, non-polar Leu and the importance of their respective locations. Similarly, in T143M mutation, a major change from polar Thr into non-polar, significantly bulkier Met within the more antigenic second loop of the *'a' *determinant occurred [41]. This is also seen when several of the substitution patterns were constructed in tertiary structure modelling (Figure 2), with more significant changes observed if the amino acids involved had higher degree of variation in their properties.

Comparison of variants T123A, M133L and T143M with the reference wild-type HBsAg showed different predicted tertiary structures with lesser degree of changes observed in the mainframe helices compared to the loops' structures (Figure 2). This might be caused by the higher degree of freedom in the movement of the loop regions. Loop regions tend to be hydrophilic and interact more freely with the surrounding environment, while mainframe helices are much more constrained in structure due to the hydrophobicity and tendency to maintain the distance between their residues [44].

All these observations were obtained by mathematical model and prediction software, involving various algorithms to calculate the antigenic index and methods to predict variant HBsAg conformation. Further analysis involving experimental studies of the interaction between variant HBsAg and anti-HBs is needed to confirm these preliminary findings, and continuous screening of larger sets of samples is necessary to obtain more data on the emergence of new variants that might circulate in the population.

## Conclusions

In conclusion, antigenic index analysis and *de novo *prediction of tertiary conformation of the three HBsAg variants (T123A, M133L, and T143M) found in Indonesian blood donor samples with undetectable HBsAg revealed that T143M substitution altered the antigenicity most significantly compared to the other two mutation patterns and the other known variants. This finding offers insight into the possibility of predicting antigenic changes in unique variants based on its primary amino acid sequence. It also underlines the importance of protein structure prediction in understanding the dynamic interactions between pathogenic agents and host immune system, in anticipation of new variants that might emerge in the future. This would in turn be a useful tool to better overcome the issues regarding detection failure by diagnostic assays and the global use of vaccines, particularly in endemic areas, as one possible mechanism of selecting escape mutants.

## Competing interests

The authors declare that they have no competing interests.

## Authors' contributions

SII carried out the protein prediction analysis, participated in the sequence alignment and drafted the manuscript. MDT carried out the molecular genetic studies, sequence analysis, and the design of the study. MR participated in the serological and molecular genetic studies. DHM conceived of the study, and participated in its design and coordination and helped to draft the manuscript. All authors read and approved the final manuscript.
